# Domestic Soft Law Regulation during the COVID-19 Lockdown in Hungary: A Novel Regulatory Approach to a Unique Global Challenge

**DOI:** 10.1017/err.2020.115

**Published:** 2021-03

**Authors:** Petra Lea LÁNCOS, László CHRISTIÁN

**Affiliations:** * Associate Professor, Péter Pázmány Catholic University; Head of Secretariat, Hungarian Constitutional Court, Budapest, Hungary; email lancos.petra.lea@jak.ppke.hu.; ** Associate Professor, Vice Rector for Education, University of Public Service, Budapest, Hungary; email: christian.laszlo@uni-nke.hu.

## Abstract

On 13 March 2020 the Hungarian Government announced the immediate closure of all schools throughout the country to prevent the spread of COVID-19 forcing several hundred thousand children to learn from home, and teachers to ensure their education. The Hungarian Educational Authority hurried to issue recommendations on the use of digital education tools. During the COVID-19 pandemic and the special legal order consequently introduced by the Hungarian Government, Hungary has seen the emergence of such non-binding measures adopted by public entities, complementing governmental action against the pandemic, with the aim of providing guidance to bodies exercising a public service function (“addressees”). These protective measures adopted under the special legal order are deemed to be successful and are largely followed by the addressees. Since soft law has hitherto been neglected by both Hungarian administrative governance and the legal literature, the recent burgeoning of non-binding measures deserves scholarly attention. In this article, we set out to map the specific context of the emergence of domestic soft law and the conditions for its adoption and reception, relying on our case study conducted in respect of the National Educational Authority’s recommendations.

## I. Introduction

Countries around the world followed different approaches in managing their response to the COVID-19 pandemic. In Hungary, the government decided to declare a state of danger on 11 March 2020, a form of special legal order under the Fundamental Law.^1^ Relying on its special legislative powers, the Hungarian Government instituted a lockdown and took action in various areas to prepare for the rise of COVID-19 cases, adapt administrative and other services to the requirements of the public health emergency and mitigate the effects of the ensuing economic downturn on businesses and employees alike.^2^ The government’s efforts were complemented by administrative authorities, bodies and agencies playing an important role in both informing the public and issuing non-binding measures for external addressees in response to new situations posed by the pandemic.

Our article focuses on the emergence of such “external” non-binding measures^3^ issued by public entities as a tool to navigate the challenges arising during the state of danger. We argue that the proliferation of domestic soft law adopted by various administrative authorities, bodies and agencies to guide the behaviour of third parties at the time of the COVID-19 pandemic is a milestone in Hungarian administrative-regulatory behaviour, since beyond instances of internal guidance,^4^ non-binding measures have hitherto hardly played a role in national administrative governance.^5^ Meanwhile, an assessment of these instances of “external” domestic soft law reveals that the strong centralising forces experienced in the Hungarian administrative system have also left their mark on the formulation and adoption of these non-binding measures.

In what follows, we first describe the stance towards soft law followed by the Hungarian courts, authorities and domestic legal scholarship in general. Next, we elaborate on the constitutional backdrop of the state of danger, and in particular, the specific social and public health context of the emergence of domestic external soft law during the pandemic. To provide insight into the adoption and implementation of these non-binding measures, we discuss a case study, focusing on the recommendations of the Hungarian Educational Authority issued to assist educational institutions in fulfilling their tasks under the lockdown and their reception by schools.^6^ To prepare the case study, we conducted an interview with Renáta Vanó, Vice President of the National Educational Authority, and conducted a small-scale survey among school principals heading public, private and church-run schools, focusing on the transparency and participation aspects of soft law regulation.

Looking at the specific recommendations issued by the National Educational Authority, we conclude that, while they had been adopted with considerable delay and schools were forced to develop alternative solutions to meet the challenge of online education, addressees remain open to such guidance. The example of the National Educational Authority’s recommendations also shows that, while the driver of recent soft law regulation is the government, based on the experiences with the recent emergence of domestic soft law, non-binding external measures exhibit hitherto untapped potential in Hungarian administrative governance.

## II. Adoption, perception and application of soft law in Hungary: a story untold

Although legal scholarship pays increasing attention to international and EU soft law,^7^ focusing on the delimitation,^8^ nature and purpose,^9^ classification^10^ and legitimacy of in particular EU soft norms,^11^ and lately, also their national implementation,^12^ the topic of domestic soft law is largely neglected in Hungarian academia.^13^ While the phenomenon of non-binding measures is not unknown in Hungary, these sources do not form part of university curricula, nor is there an abundance of scholarly work detailing their taxonomy or application.^14^

Non-binding measures such as ministerial circulars, ethical codes for public servants or judges, notices of the Competition Authority or opinions and resolutions of the Curia (the Supreme Court of Hungary) are, however, routinely issued in Hungary. Yet these are typically self-binding acts,^15^ addressing elements and issues within the respective organisation (eg Curia opinions) or informing the public about the Authority’s intended approach in a given matter (eg the Hungarian Competition Authority’s notice on fines). Meanwhile, external soft law, ie non-binding measures issued by public entities to provide guidance to parties outside their organisation, are extremely rare. This may be due to the system of norms regulated under Act No CXXX of 2010 on law-making. According to this Act, a regulator may adopt two main types of norms:^16^ legal acts with general force (subject to restrictions on personal, territorial, temporal and material scope),^17^ and so-called “public law regulatory instruments”, which include self-binding acts.^18^ The general binding force of legal acts is established in Article R(2) and Article T(1) of the Fundamental Law.^19^ Meanwhile, it is Article 23 of the Act on law-making that details the addressees of public law regulatory instruments, adopted “to regulate the organisation, operation and activities of organs under [the issuing institution’s] control, direction or supervision”, which “shall be binding upon the personnel of the organ concerned”.^20^ Accordingly, these norms are binding measures, albeit only on the issuing body’s organisation.

In her pioneering work on “pseudo norms” (as she refers to soft law issued by Hungarian public entities), Géczi underlines that, while constitutionally^21^ all law-making requires a legal basis, non-binding measures may be freely adopted.^22^ This regulatory leeway notwithstanding, authorities, bodies and agencies typically restrict themselves to issuing only self-binding measures, perhaps as a spill-over effect from the limited scope of public law regulatory instruments. Another reason could be the more recent and increasing centralisation taking place in the Hungarian administrative system, reinforcing the disinclination to experiment with alternative regulatory solutions.

To gauge the perception and application of domestic soft law, we traced the relevant judicial practice by conducting keyword searches in the electronic database operated by the National Office for the Judiciary.^23^ The database, called the Compendium of Court Decisions, comprises anonymised judgments, decisions, and opinions rendered by Hungarian courts since 1 July 2007. In the framework of our keyword search, we sifted through decisions rendered in 2020 including the keywords “recommendation”, “circular” and “communication”, excluding hits involving EU soft law measures and other uses of these keywords not referring to non-binding measures adopted by public entities. Based on the results, it seems that courts consider domestic soft law as a complementary source, referring to it in order to bolster an argument,^24^ but otherwise, the courts insist on their non-binding nature.^25^ For example, the Curia highlighted in its judgment Kf.II.37.119/2019/5 that recommendations of the Hungarian Financial Supervisory Authority “are not legal acts and are not exempt from compliance with [the law]”. Meanwhile, in its judgment Pvf 20094/2019/12, the Curia recognised the self-binding character of the Hungarian Financial Supervisory Authority’s recommendations. These preliminary findings show that Hungarian courts will consider domestic soft law if deemed relevant to the case, in particular, to give further weight to the reasoning in support of applicable law or other relevant facts of the case. Nevertheless, they only recognise the self-binding character of such norms and exclude all forms of *contra legem* application of domestic soft law.

Based on the above, domestic soft law regulation remains an uncharted territory of Hungarian legal scholarship, while regulators also seem disinclined to resort to this type of rule-making. As far as the courts are concerned, soft law is used merely to bolster existing legal arguments, and is consistently deemed non-binding where it runs counter to “hard” law.

## III. The administrative, constitutional and regulatory context of emerging domestic soft law

In what follows, we set the scene for the recent emergence of domestic soft law issued by authorities, agencies and bodies. We briefly describe the broader context of the incremental centralisation of the Hungarian administrative system as the backdrop for all administrative-regulatory activity. Then we move on to the immediate constitutional context of the special legal order under the state of danger introduced by the government in response to the COVID-19 threat. Finally, we provide a short overview and typology of regulatory efforts undertaken with the aim of meeting the challenges posed by the pandemic, highlighting the role of “external” soft law within the regulatory framework.

### 1. Centralisation in the Hungarian administrative system

In the past decade, the Hungarian administrative system underwent considerable transformation under the governmental strategy of building a “good state”.^26^ In Szigetvári’s words, “a completely different governance model was created, with a strong, neo-Weberian state that has been expanding at the cost of local elected governments (…) The primary goal of centralisation is to increase the efficiency and effectiveness of the administrative system.”^27^

Under the “good government paradigm” and the concept of “strong state”,^28^ the government transformed the system of regional administration by introducing the district system.^29^ In parallel to reducing the competencies of regional administrative bodies and local self-governments, the so-called government offices in the capital and the counties were increasingly furnished with more powers. The government wanted to bring administration as close to the citizens as possible, employing competent public servants to ensure speedy and effective administration. Szigetvári points out:

the systemic reforms outlined in the (…) Hungarian administrative reform program (*Magyary Program*) launched after 2010 is on the belief that a public administration with stronger ties to the central government has a greater professional competence and/or stronger loyalties to politicians than public servants working in decentralised organisations (agencies, local governments).^30^

Government office employees were therefore enrolled in a comprehensive central training programme and finally, in 2013, a total of 198 district offices were opened.^31^

Since 2014, integrated one-stop-shop government offices have been sprouting up throughout the country. Specialist administrative services (eg social services, health insurance, pension insurance, labour administration) were integrated under the government and district office system. This transformation of the Hungarian administrative system led to a more centralised, hierarchical administrative structure.^32^

### 2. State of danger as a form of special legal order

Recognising the public health threat posed by the COVID-19 disease, the Hungarian government took various actions to prevent its transmission and to prepare for a possible epidemic, as well as to meet the economic, educational and administrative challenges posed by the ensuing lockdown. In light of the rapidly spreading virus, the government established the Operational Corps responsible for the containment of the COVID-19 infection,^33^ whose tasks included the monitoring and evaluation of the spread of the virus, consultation, the introduction of epidemiological measures and informing the public. The Minister for the Interior and the Minister for Human Capacities^34^ were appointed to head the new Operational Corps. Together with further members of the Operational Corps,^35^ it is clear that, in addition to health and epidemiological expertise, the government made law enforcement bodies, in particular the police and the defence forces, an integral part of pandemic management. This stance is further reflected in the appointment of so-called hospital commanders and commanders of critical undertakings,^36^ as well as in the designation of critical infrastructure. This law-enforcement-heavy approach strongly influenced the institutional and procedural aspects of the fight against the epidemic, providing for a stringent, hierarchical, centralised and “militant” context for national epidemic management.

In light of the spreading of COVID-19 in Europe, the government took the decision based on Article 53 of the Fundamental Law to declare the so-called “state of danger” on 11 March 2020.^37^ According to Article 53 of the Fundamental Law:

(i) In the event of a natural disaster or industrial accident endangering life and property, or in order to mitigate the consequences thereof, the Government shall declare a state of danger, and may introduce extraordinary measures laid down in a cardinal Act.^38^
- (ii) In a state of danger the Government may adopt decrees by means of which it may, as provided for by a cardinal Act, suspend the application of certain Acts, derogate from the provisions of Acts and take other extraordinary measures.
- (iii) The decrees of the Government referred to in Paragraph (2) shall remain in force for 15 days, unless the Government, on the basis of authorisation by the National Assembly, extends those decrees.
- (iv) Upon the termination of the state of danger, such decrees of the Government shall cease to have effect.

The state of danger is a form of special legal order established under the Fundamental Law (just like the “state of national crisis”,^39^ the “state of emergency”,^40^ the “state of preventive defence”,^41^ the “state of terrorist threat”^42^ and the “unexpected attack”^43^). The rules governing the state of danger authorise the government to govern by decree, accelerating the decision-making process. At the same time, they allow for the suspension or restriction of most fundamental rights (with the exception of the right to life, human dignity, the prohibition of torture, inhumane or degrading treatment and the presumption of innocence) without the safeguards of proportionality or the preservation of the essential content (*Kerngehalt*) of such rights. According to the Hungarian Fundamental Law, such governmental decrees are to remain in force for only 15 days.^44^ A few days before the lapse of the extraordinary COVID-19 measures, the government introduced a bill in Parliament to extend the effect of all protective measures beyond the 15-day deadline until the end of the state of danger. This controversial bill was passed into law by the Parliament, relieving the government of the obligation to reinstate the same measures every 15 days.^45^

### 3. Regulatory efforts to contain the spread of COVID-19

The challenges posed by the pandemic were met with a specific regulatory mix including laws adopted by the Parliament, a number of government decrees and decisions and an array of non-binding measures issued by authorities, agencies and bodies.

The government adopted various binding decrees and decisions geared towards ensuring the safety of the population (eg closing down Hungarian borders, as well as non-essential retail shops and entertainment facilities; travel restrictions^46^) and the mitigation of the economic consequences of the lockdown (eg wage subsidies^47^). New technologies were also harnessed to contribute to the fight against the epidemic: Governmental Decree No 181/2020 (V 4) authorised the use of electronic means of controlling quarantine measures, with the police gathering data from an application (*VírusRadar*) voluntarily installed by those who had been put under quarantine with police supervision. With the help of the app, authorities gain access to the user’s profile, movements and health data, processing it for a maximum of 60 days following the lapse of the individual’s quarantine.

In a more controversial move, a Parliamentary amendment to the law governing the National Security Service authorised the Service to control the electronic communication traffic of state and municipality bodies to avert a possible cyber-attack, without, however, providing guarantees for the protection of personal data.^48^ The Parliament complemented the prohibition on scaremongering in the Penal Code with a paragraph designed for the specific context of the special legal order,^49^ which was widely criticised as possibly restricting freedom of speech.^50^

Certain authorities and agencies, such as the National Health Care Centre,^51^ the Hungarian Tourism Agency,^52^ and the National Educational Authority^53^ took the lead in issuing recommendations and guidance on topics from sanitation and remote education to the operation of restaurants during the pandemic. Several of these recommendations were based on guidelines from WHO or other international organisations.

Finally, on 16 June 2020 the Hungarian Parliament called upon the government via Act No LVII of 2020 to abolish the state of danger, which the government did by decree on the following day.^54^ Some restrictions regarding quarantine, travel, the mandatory wearing of masks in shops and on public transport, bans on events over a certain size and easements such as moratoriums on loan repayments are still in place.^55^ As far as education is concerned, at present the government plans to reopen schools for the fall/winter semester, with institutions of higher education left to determine individually how they wish to continue teaching (remotely, in-person or using hybrid forms of teaching). Nevertheless, should the closure of schools be initiated as part of any future lockdown, the National Educational Authority’s recommendations are meant to provide guidance for remote education.

## IV. Case study: the adoption and reception of the National Educational Authority’s recommendations

This section discusses our case study, where we analyse the adoption and reception of the external soft law issued by the National Educational Authority during the lockdown. Following a brief introduction to the National Educational Authority’s tasks and competences, we take a look at the legal basis for the adoption of the Authority’s non-binding measures. Next, we present the findings of our interview conducted with the Vice President of the National Educational Authority concerning the conditions surrounding the adoption of the external soft law issued under the lockdown, and finally we turn to the results of the survey on the reception and implementation of these measures sent out to a dozen Hungarian nursery schools and schools.

### 1. The National Educational Authority and the legal basis for issuing external soft law

The National Educational Authority was established in 2013 as a central authority subordinated to the minister responsible for education.^56^ It coordinates all areas of training and education in Hungary, with activities ranging from registration and certification through administration of applications to secondary and tertiary education institutions, as well as the recognition of foreign diplomas and qualifications to the provision of information. It collects and records data related to education from nursery schools, elementary and secondary schools, universities and training centres. It issues student IDs, conducts nation-wide student assessments and measures teachers’ performance. Finally, the Authority is responsible for licensing language examination centres and plays a part in organising national student competitions.^57^

An important aspect of the National Educational Authority’s work is to support the work of educational establishments. The day after the government announced the closure of schools, it adopted Government Decision No 1102/2020 (III. 14) on the introduction of a new work schedule in public education and vocational training institutions due to COVID-19. According to this Government Decision, out-of-classroom education was to be organised in schools accompanied by the digital monitoring and support of the learning process. Article 1(c) established the legal basis for the external soft law measures analysed in this study. Without naming the responsible institution, the Government Decision set forth “a methodological recommendation is issued to implement [the out-of-classroom education]”. It was the National Education Authority that finally adopted these methodological recommendations on 9 April 2020, comprising the following instruments: the “Methodological recommendation for out-of-class digital work schedule”;^58^ the “Methodological recommendations for kindergarten, arts and other special education situations”;^59^ and the “IT security recommendation for teaching and learning in a digital work schedule outside the classroom”^60^ and published them on its website.

The recommendations are addressed to nursery schools, elementary and secondary schools and other teaching facilities and institutions. That is, these recommendations are addressed to institutions outside the organisation of the National Educational Authority. These measures do not bear the formal characteristics of legal acts: they have no title number, sections or numbered paragraphs. While their titles include the word “recommendation”, which clearly indicates their non-binding nature as far as their wording is concerned, strongly exhortative phrasing such as “we ask teachers to”, “should prepare for”, “provide students with”, etc^61^ is used. Since the recommendations are not legal acts, or internal guidance addressed to institutions within the organisation’s hierarchy, the mandatory wording of the measures notwithstanding, the recommendations can be classified as external soft law measures.

### 2. Circumstances surrounding the adoption of the National Educational Authority’s recommendations

Since there is no information in formal law or on the website of the National Educational Authority describing the legal basis, process and adoption of the recommendations, we decided to contact the Authority. In order to gain insight into the circumstances surrounding the elaboration of the analysed recommendations, in particular into the transparency and participation-related aspects of the adoption process, we conducted an interview with the Vice President of the National Educational Authority on 31 July 2020.^62^ Our questions focused on the legal basis of the recommendations, whether the Authority involved stakeholders in the elaboration of the substance of the recommendations, and the possible monitoring of their implementation.

In the interview, the Vice President of the National Educational Authority confirmed that the Authority had not issued external soft law before the state of danger. The closure of schools during the COVID-19 lockdown, however, was a unique situation that made the adoption of guidance addressed to educational institutions necessary. Schools were obliged to ensure ongoing teaching based on the National Core Curriculum and to prepare students for final secondary school examinations. The challenge was that out-of-classroom teaching had to be organised through online means and to be determined and implemented individually by the schools themselves. The online tool developed and made available previously by the state was the so-called KRÉTA platform, which could be used for recording students’ grades, publishing homework, assignments and conducting teacher-to-student/parent correspondence. Schools were free to choose the online tools available, including KRÉTA but also other platforms, such as Zoom, MSN Teams, Google Classroom, EduBase, etc. It was in respect of the online teaching activity that the National Educational wished to provide support with its recommendations.

The recommendations were issued on the basis of Article 1(c) of Government Decision No 1102/2020 (III. 14), with the “permission and approval” of the Ministry of Human Capacities, meaning that the task of elaborating the recommendations was delegated to the National Educational Authority and internally signed off on by the Ministry. Apart from calling on the competent ministry to prepare the methodological recommendation, the Government Decision did not set forth any substantive points on what these recommendations should contain. The recommendations were elaborated with input from the competent ministry, public education institutions and the bodies operating public schools, as well as pedagogical services. Following their adoption, the recommendations were published in mid-April on the National Educational Authority’s website where they are freely accessible. As of yet, the National Educational Authority has not modified the recommendations, nor has it monitored their implementation or requested feedback on the same.

Based on our interview, we conclude that the recommendations analysed for this article were not adopted by the National Educational Authority *ex proprio motu*, but were based on the legal basis contained in Government Decision No 1102/2020 (III. 14). Moreover, they were issued with the permission and approval of the competent ministry. Therefore, no legal or constitutional limits have been overstepped in the adoption of these recommendations and as far as the transparency and participation aspects of the measures are concerned, the National Educational Authority informed us that they relied on expert knowledge, such as public education institutions and pedagogical services.

### 3. Reception of the National Educational Authority’s recommendations by public, private and church-run schools

To gauge the reception of the National Educational Authority’s recommendations, we prepared a survey, which we sent out to the principals of a dozen schools and nursery schools in July and August 2020. To ensure that our findings reflect the institutional diversity in Hungary, we sent out the survey to public, private and church-run schools and nursery schools headed by principals among our personal contacts and among contacts who were recommended to us. Unfortunately, no nursery schools responded; however, we received responses from public (one), private (two) and church-run schools (one), amounting to four responses in total. As such, the responses received are relevant only to the recommendations addressed to schools and we have no information related to the reception of “Methodological recommendations for kindergarten, arts and other special education situations”.^63^ While, based on the number of responses received, we cannot consider the results of our mini-survey to be in any way representative, they nevertheless give some insight into the reception of the recommendations analysed.

Our first group of questions related to procedural aspects of the adoption of the recommendations, mainly, the aspects of transparency and participation:

Did you as a school principal seek assistance from the National Educational Authority that they provide guidance on the adjustment to the new situation? Do you perhaps know any school principals who had requested such guidance, assistance?

Were you, as school principal involved in any way in the elaboration of the recommendations (eg electronic consultation)?

Did you receive a separate notification about the publication of the recommendations or were you only informed about them on the National Educational Authority’s website?

While all of the school principals answered that they had not sought guidance from the National Educational Authority, they had been informed about other schools that had turned to the Authority for help. Since the closure of schools was announced on a Friday and teaching had to continue uninterrupted on the following Monday, schools had to determine the relevant “digital” teaching methods within an extremely short window of time. As the principal of the respondent public school noted, “there was no chance of asking for help over the weekend anyway”.^64^ Meanwhile, both private school respondents explained that they had already put in place the digital teaching tools before the state of danger was declared, having merely to transition to using them over the weekend.

The principals unanimously noted that they had not been involved in the elaboration of the recommendations, with one respondent heading a private school noting that they would have been open to providing input. As far as receiving specific information on the fact that the National Educational Authority had published these non-binding measures, the respondents noted that they were made aware only through Authority’s website, although the principal of the church-run school subsequently also received an email pointing them towards the recommendations.

Next, we explored the legitimacy and effectiveness of the Authority’s recommendations, to understand whether these soft law measures were considered by the addressees to be useful and acceptable guidance. To this end, we posed the following questions in our survey:

Did you implement these recommendations?

If so, was it because you found them convincing, *or* because they were issued by the National Educational Authority and as such, should be followed, *or* because they contained such general suggestions that all institutions would have been implemented anyway *or* other (explain)?

If not, was it because the recommendations are non-binding and as such do not have to be taken into account, *or* because they are not applicable to your specific institution *or* other (explain)?

Did you find these recommendations useful?

Do you agree that the National Educational Authority should provide professional support to educational institutions through non-binding measures in the future?

Would you like to provide professional input into the National Educational Authority’s recommendations?

As to the implementation of the recommendations, all principals answered that they had not implemented them, partly because they were issued too late into the lockdown and partly because they were not applicable to the systems already put in place. Since the schools were bound to transition to digital teaching mid-March, the recommendations issued mid-April were no longer considered relevant by the respondent schools. As the principal of the respondent public school noted, “the recommendations came with considerable delay and contained very general advice”,^65^ and since the school had already implemented its own solution to digital teaching by that time, they did not want to disrupt the system put in place by implementing the solutions suggested in the recommendations. Nevertheless, the recommendations were confirmation to most principals that they were “on the mark” with their solutions.^66^ Several respondents noted that the recommendations were too generally framed and were difficult to adapt to the diverse situations in which the different (public, private or church-run) schools and the affected students and families found themselves when the closures were announced. For example, in many families the lack of necessary internet connection, computers or other smart devices at home made digital learning impossible. At the same time, the majority of principals would welcome such recommendations in the future and would be happy to be involved in elaborating their substance by sharing their experience and expertise.

Finally, we asked our respondents if they had been contacted to assess whether they had implemented the recommendations of the National Educational Authority. The principals unanimously answered that no one had requested information on the implementation of these measures in their schools. This means that without a systematic evaluation of the implementation of the recommendations, the National Educational Authority has no feedback on the regulatory performance and the level of implementation of its measures.

## V. Conclusions

During the special legal order introduced to prepare for, and combat, the COVID-19 pandemic, statutes of Parliament, as well as government decrees and decisions, were complemented by a novel regulatory solution: “external” domestic soft law. These types of non-binding measures have to date hardly ever featured in the Hungarian administrative-regulatory toolbox, yet the unique challenges posed by the pandemic and the ensuing lockdown meant that there was a need to provide guidance to businesses, bodies carrying out public service functions and schools on how to adjust their operation to deliver ongoing services both safely and effectively. In view of the recent burgeoning of domestic soft law in Hungary, our article represents a first step in mapping the regulatory context, adoption and reception of these non-binding measures.

As far as the regulatory context is concerned, the Hungarian administrative system underwent considerable centralisation during the past decade, and the ensuing strict hierarchical structure was exacerbated by the requirements of the special legal order. Accordingly, the external soft law emerging in this period was not the result of regulatory experimenting, but much rather a flexible response to a regulatory obligation set forth in a government decision. As far as the recommendations analysed in our case study are concerned, their non-binding character notwithstanding, these soft norms were issued by the National Educational Authority with the approval of the competent ministry. This may be explained by the fact that the government decree specifically prescribed the adoption of these measures, for which the competent ministry was responsible. Based on our interview with the Vice President of the National Educational Authority, the recommendations were elaborated in consultation with professional bodies ensuring the necessary expertise; however, important stakeholders, such as schools or nursery schools, were not involved in developing the substance of these norms. These findings show that, at least under the special legal order (implemented because of the declared state of danger linked to COVID-19), there is a clear intention to move towards more flexible, novel regulatory solutions to emerging technical challenges, without however upsetting the strict hierarchical framework of the domestic administrative system.

Although the findings of the case study show that in the concrete case the recommendations were issued with considerable delay and with an overly general substance, respondents to our survey were unanimous in noting that such non-binding measures of the competent administrative body would be welcomed in the future. Respondents further stressed that, owing to the very different endowments and circumstances of Hungarian schools and students, future non-binding measures should be framed with a view to accommodating this diversity and providing concrete guidance to specific situations, preferably involving and building on the expertise of the affected schools themselves. While our mini-survey may in no way be considered representative in nature, it clearly indicates an openness of external addressees towards receiving guidance from professional public bodies and a willingness to participate in their elaboration, both to share best practices and to develop detailed recommendations specific to the different circumstances of the addressee institutions.

As a general conclusion, we suggest that, while the example of the National Educational Authority’s recommendations shows that the driver of recent soft law regulation was the government, based on experiences with the recent emergence of domestic soft law, non-binding external measures still exhibit hitherto untapped potential in Hungarian administrative governance. Beyond their flexibility in time-sensitive situations, the appeal of such non-binding norms may be that within the framework of the highly centralised Hungarian administrative system, external soft law can exert a strong compliance-pull towards addressees.^67^ Developing external domestic soft law also outside the period of the special legal order would be a welcome addition to the existing regulatory toolbox of domestic decision-makers, in particular, if stakeholders were involved in developing their substance. On this basis, external soft law has the potential to become an important regulatory instrument in Hungarian administrative governance, ensuring useful professional guidance to domestic stakeholders.

## Footnotes

1 Hungarian Constitution 2011.

2 Based on the number of cases, and in particular, the relatively low number of deaths, the fight against the pandemic at its current stage may be considered successful; see Johns Hopkins University & Medicine <coronavirus.jhu.edu/map.html> (last accessed 18 August 2020).

3 Cf LA Jiménez and JM Rodríguez de Santiago, “European and domestic soft law within Spanish administrative law” (2020) 2(20) Preprints series of the Center for European Studies Luis Ortega Álvarez <blog.uclm.es/catedrajeanmonnet/files/2020/01/PrePrint2-20.pdf> (last accessed 18 August 2020).

4 ibid.

5 Instances of external soft law measures include eg the recommendations of the Hungarian Financial Supervisory Authority, whose tasks, as a result of centralisation, were integrated into the Hungarian National Bank (Magyar Nemzeti Bank) <www.mnb.hu/archivum/Felugyelet/root/fooldal/bal_menu/szabalyozo_eszkozok/pszafhu_bt_ajanlirelvutmut/pszafelnoke_ajanlasai> (last accessed 18 August 2020).

6 We focused on this topic because, of the public bodies adopting external domestic soft law during the lockdown, only the National Educational Authority was open to answering our questions.

7 Eg as early as 1957 László Buza wrote about programme-like norms when referring to what is now called international soft law: L Buza, “A törvényesség és igazságosság elve a nemzetközi jogban” (1957) 3 Acta Universitatis Szegediensis de Attila József 1. For more recent works, see in particular F Snyder, “The Effectiveness of European Community Law: Institutions, Processes, Tools and Techniques” (1993) 56 MLR 1; AT Guzman and TL Meyer, “International Soft Law” (2010) 2 J Leg Anal 1.

8 Eg A Peters and I Pagotto, “Soft Law as a New Mode of Governance: A Legal Perspective. New Modes of Governance Project” (2006) <www.eu-newgov.org/database/DELIV/D04D11_Soft_Law_as_a_NMG-Legal_Perspective.pdf> (last accessed 18 August 2020); OA Ştefan, “European Union Soft Law: New Developments Concerning the Divide Between Legally Binding Force and Legal Effects” (2012) 75 MLR 5, 879, 879.

9 GM Borchardt and KC Wellens, “Soft Law in European Community Law” (1989) 14 EL Rev 5; L Senden, *Soft Law in European Community Law* (Hart 2004); A Peters, “Soft Law as a New Mode of Governance”, in U Diedrichs et al (eds), *The Dynamics of Change in EU Governance* (Edward Elgar 2011).

10 F Terpan, “Soft Law in the European Union – The Changing Nature of EU Law” (2015) 21 ELJ 1; A Peters, “Typology, utility and Legitimacy of European Soft Law” in A Epiney et al (eds), *Die Herausforderung von Grenzen: Festschrift für Roland Bieber* (Nomos 2007); DM Trubek et al, “‘Soft Law’, ‘Hard Law’, and European Integration: Toward a Theory of Hybridity” (2015) Univ of Wisconsin Legal Studies Research Paper 1002.

11 M Hartlapp, *Soft Law Implementation in the EU Multilevel System: Legitimacy and Governance Efficiency Revisited* (Springer 2019).

12 See in particular the publications of the SoLaR network, including M Eliantonio et al, “EU Environmental Soft Law in the Member States: A Comparative Overview of Finland, France, Germany, Italy, the Netherlands, Slovenia and the UK” (2020) <papers.ssrn.com/sol3/papers.cfm?abstract_id=3656418> (last accessed 18 August 2020).

13 There are exceptions, eg PL Láncos, “The approach of Hungarian authorities to soft law: On the road to where?” forthcoming in M. Eliantonio et al (eds), *EU Soft Law in the Member States: Theoretical Findings and Empirical Evidence* (Bloomsbury), or J van der Sluijs focuses on non-binding measures issued by private entities: “The Infrastructure of Normative Legitimacy in Domestic Soft Law – Sketching the Field” (2017) 62 Sc St L.

14 Exceptions include K Géczi’s paper “A közigazgatási büntetőjog szabályozási anomáliái, különös tekintettel a pszeudo normák kérdéskörére” (2009) Jog – Állam – Politika 3, 177, and Attila Kun’s paper on soft law in the ambit of labour law, with a strong focus also on domestic soft law, in particular, non-binding measures issued by stakeholders: A Kun, “A puha jog (soft law) szerepe és hatékonysága a munkajogban − Az új Munka Törvénykönyve apropóján” (2012) <plwp.eu/docs/wp/2012/2012-41-Kun.pdf> (last accessed 18 August 2020).

15 Cf Géczi, supra, note 14, p 187.

16 Art 1(1) of Act No CXXX of 2010 on law-making <njt.hu/translated/doc/J2010T0130P_20180518_FIN.pdf> (last accessed 3 August 2020). See also Géczi, supra, note 14, p 182.

17 Arts 6–7 of Act No CXXX of 2010 on law-making.

18 Arts 23–25 of Act No CXXX of 2010 on law-making.

19 Art R(2) of the Fundamental Law provides: “The Fundamental Law and *the laws* shall be binding on everyone”. Art T(1) of the Fundamental Law foresees: “Generally binding rules of conduct may be laid down in the Fundamental Law or *in laws*, adopted by an organ having legislative competence and specified in the Fundamental Law, which are promulgated in the official gazette” (added emphasis).

20 Art 37(2) of Act No CLI of 2011 on the Constitutional Court, see further ÍE Horváth et al, Közigazgatási perjog (Dialóg Campus 2019) pp 139–40.

21 Art T of the Fundamental Law of Hungary.

22 Géczi, supra, note 14, p 183.

23 Compendium of Court Decisions (Bírósági Határozatok Gyűjteménye) <birosag.hu/birosagi-hatarozatok-gyujtemenye>. We found a total of four references: one circular and three recommendations of the Hungarian Financial Supervisory Authority in these judgments: 26.B.288/2011/23, Kf.II.37.199/2019/5, Pvf.2009/2019/12 and 16.Gf.40.026/2020/711.

24 Eg in judgment 26.B.288/2011/231 of the Budapest-Capital Regional Court and judgment 16.Gf.40.026/2020/7 of the Metropolitan Regional Court.

25 Of course, where the recommendation stems from the Curia itself (the Supreme Court of Hungary), courts refer to, and comply with its substance since these are self-binding norms, Cf Decision Nos 10.Gf.40.601/2019/16, 19.Gf.40.236/2019/19 and Gf.III.30.025/2020/5 of the Metropolitan Regional Court; Gf.III.30.025/2020/5.

26 On the meaning of the “good state” and the *Magyary programme*, see G Balogh, “Gondolatok a magyar közigazgatás reformjáról” (2015) 8 Új magyar közigazgatás 4, 26 et seq.

27 T Szigetvári, “The Hungarian Administrative System: From Centralization to Regionalization and Back” (2020) 3 JPAL 1, 37.

28 I Stumpf, *Reinventing Government and Constitutional Changes in Hungary* (Gondolat 2017) p 2.

29 Act No XCIII of 2012 on the establishment of the district system and the amendment of related laws.

30 Szigetvári, supra, note 27, p 37.

31 Act CXLIX of 2010 on the amendment of the Act CVII of 2004 on the Associations in Small Regions.

32 See in detail Stumpf, supra, note 28, pp 68–70.

33 Government decision No 012/2020 (I. 31) on the establishment of the Operational Corps responsible for the containment of COVID-19 infection.

34 The so-called Ministry for Human Capacities was established in 2010 after merging the individual ministries responsible for education and culture, health, social and labour affairs, and the sports department of the Ministry for Self-government.

35 Members of the new body included the Chief Medical Officer, the Director General for Public Safety and the National Police Chief, as well as the directors of the National Directorate General for Disaster Management, the National Directorate General for Aliens Policing, the Counter-Terrorism Centre, the South-Pest Hospital Centre, the State Health Care Centre and the National Ambulance Service.

36 A total of 51 hospital commanders were tasked with enforcing rules adopted to combat the epidemic and to ensure the safeguarding of medical supplies. The Task Force for the Security of Critical Hungarian Undertakings prepared for the operation and protection of critical undertakings. Website of the Hungarian Government <www.kormany.hu/hu/honvedelmi-miniszterium/hirek/az-orvosigazgatokat-tehermentesitik-a-korhazparancsnokok> (last accessed 4 August 2020).

37 Government Decree No 40/2020 (III. 11) on the promulgation of the state of danger. Since the change of political regime in Hungary in 1989 a state of danger has been declared only three times, in 2001 at the time of the flooding of the Tisza river, in 2010 following an industrial accident leaking liquid waste around Ajka, and finally in 2013 during the flooding of the Danube river.

38 Cardinal laws (*sarkalatos törvények*) are legal acts adopted or amended by the Hungarian Parliament by a two-thirds majority that typically, although not exclusively, regulate the details of key constitutional institutions, such as Act No CXXX of 2010 on law-making. The cardinal law referred to in the Fundamental Law, Art 53 is Act No CXXVIII of 2011 concerning disaster management and amending certain related acts. On the history of laws requiring a two-thirds majority in Hungary, and on the origin of the designation *sarkalatos törvény*, the risks of calcification or, vice versa, the Parliamentary minority protection function of cardinal laws, see H Küpper, “A kétharmados/sarkalatos törvények jelensége a magyar jogrendben” (2014) MTA Law Working Papers 46.

39 Art 49 of the Fundamental Law.

40 Art 50 of the Fundamental Law.

41 Art 51 of the Fundamental Law.

42 Art 51/A of the Fundamental Law.

43 Art 52 of the Fundamental Law.

44 See also: European Parliamentary Research Service, “States of emergency in response to the coronavirus crisis: Situation in certain Member States” (June 2020) <www.europarl.europa.eu/RegData/etudes/BRIE/2020/649408/EPRS_BRI(2020)649408_EN.pdf> (last accessed 24 September 2020).

45 Act No XII of 2020 on the fight against the coronavirus was widely criticised within and outside of Hungary as giving blanket authorisation to the Government to “govern indefinitely through protective measures”. In fact, the Parliament may revoke the authorisation even before the end of the state of danger, and it is up to the Parliament to repeal the Act when the emergency situation ends. Of course, with a super-majority, the Government was not facing any threats to its powers in Parliament.

46 Eg Government Decree Nos 341/2020 (VII. 12) and 291/2020 (VI. 17) on travel restrictions during the epidemiological preparedness period.

47 Eg Government Decree No 290/2020 (VI. 17) on certain issues relating to wage subsidies for the purposes of job protection.

48 Amendment to Act No XXXI of 2020 on the amendment of certain laws to improve the safety of citizens.

49 The new para (2) of Art 337/A of the Penal Code foresees a harsher penalty for those, who “during the period of special legal order and in front of a large audience, states or disseminates any untrue fact or any misrepresented true fact that is capable of hindering or preventing the efficiency of protection”.

50 See eg G Polyák, “‘Enabling act’ in Hungary: uncontrolled government power, threatened press” (2020) Internet Policy Review <policyreview.info/articles/news/enabling-act-hungary-uncontrolled-government-power-threatened-press/1466> (last accessed 24 September 2020); C Győry, “Fighting Fake News or Fighting Inconvenient Truths? On the Amended Hungarian Crime of Scaremongering” (2020) <verfassungsblog.de/fighting-fake-news-or-fighting-inconvenient-truths/> (last accessed 24 September 2020); for the opposing view, see A Koltay, “Coronavirus and freedom of speech in a state of danger” (25 March 2020) <abouthungary.hu/blog/coronavirus-and-freedom-of-speech-in-a-state-of-danger/> (last accessed 8 August 2020).

51 Eg Recommendation on drinking water supply, sewage treatment, hygiene and waste management during the COVID-19 epidemic (29 April 2020, based on the WHO’s Interim Guidance on Water, Sanitation, Hygiene (WASH) and waste management for the prevention of COVID-19); Recommendation on the reopening of the terraces and patios of restaurants (19 May 2020); Recommendation for the reinstallation of the water system in decommissioned buildings (5 June 2020).

52 The public National Tourism Agency (governed by Government decree No 61/2017 (III. 20) on the tasks of the Hungarian Tourism Agency, a private limited company related to tourism and hospitality) issued the COVID 19 Handbook for the operators of tourist attractions and service providers (4 May 2020) including recommendations and guidance.

53 Methodological recommendation for digital work outside the classroom; IT security recommendation for teaching and learning outside the classroom in a digital work schedule (9 April 2020).

54 Government Decree No 282/2020 (VI 17) on abolishing the state of danger declared on 11 March 2020.

55 Act No LVIII of 2020 on the transitional rules related to abolishing the state of danger and epidemiological preparedness.

56 Government Decree No 121/2013 (IV.26) on the National Educational Authority.

57 O Hivatal,“Tasks of the Office” <www.oktatas.hu/hivatal/a_hivatal_feladatai> (last accessed 24 September 2020).

58 See <www.oktatas.hu/kozneveles/ajanlas_tantermen_kivuli_digitalis_munkarendhez> archived at the Wayback Machine (web.archive.com).

59 Originally published: <www.oktatas.hu/kozneveles/ajanlas_tantermen_kivuli_digitalis_munkarendhez/ovodai_ami_sni_modszertani_ajanlasok> 9 April 2020 (irretrievable).

60 Originally published: <www.oktatas.hu/kozneveles/ajanlas_tantermen_kivuli_digitalis_munkarendhez/informatikai_biztonsag> 9 April 2020 (irretrievable).

61 See <www.oktatas.hu/kozneveles/ajanlas_tantermen_kivuli_digitalis_munkarendhez>, archived at the Wayback Machine (web.archive.com).

62 Before the interview, we contacted the Vice President of the National Educational Authority outlining our research, the main issues we would discuss in the interview and the journal in which the article would be published. Ethical approval was not needed for this empirical work.

63 Originally published: <www.oktatas.hu/kozneveles/ajanlas_tantermen_kivuli_digitalis_munkarendhez/ovodai_ami_sni_modszertani_ajanlasok> 9 April 2020 (irretrievable).

64 Unpublished survey results, on file with authors.

65 Unpublished survey results, on file with authors.

66
ibid.

67 Cf Géczi, supra, note 14, p 186.

